# Calcium‐dependent activation of transglutaminase 2 by nanosecond pulsed electric fields

**DOI:** 10.1002/2211-5463.12227

**Published:** 2017-06-09

**Authors:** Keiko Morotomi‐Yano, Ken‐ichi Yano

**Affiliations:** ^1^ Department of BioelectricsInstitute of Pulsed Power ScienceKumamoto UniversityJapan

**Keywords:** calcium, nanosecond pulsed electric field, protein‐protein crosslinking, transglutaminase 2

## Abstract

Exposure of cultured human cells to nanosecond pulsed electric fields (nsPEFs) elicits various cellular events, including Ca^2+^ influx and cell death. Recently, nsPEFs have been regarded as a novel physical treatment useful for biology and medicine, but the underlying mechanism of action remains to be fully elucidated. In this study, we investigated the effect of nsPEFs on transglutaminases (TGs), enzymes that catalyze covalent protein modifications such as protein–protein crosslinking. Cellular TG activity was monitored by conjugation of cellular proteins with biotin‐cadaverine, a cell‐permeable pseudosubstrate for TGs. We applied nsPEFs to HeLa S3 cells and found that overall catalytic activity of cellular TGs was greatly increased in a Ca^2+^‐dependent manner. The Ca^2+^ ionophore ionomycin significantly augmented nsPEF‐induced TG activation, further supporting the importance of Ca^2+^. Among human TG family members, TG2 is known to be the most ubiquitously expressed, and its catalytic activity requires elevated intracellular Ca^2+^. Given the requirement of Ca^2+^ for TG activation by nsPEFs, we performed depletion of TG2 by RNA interference (RNAi). We observed that TG2 RNAi suppressed the nsPEF‐induced TG activation and partially alleviated the cytotoxic effects of nsPEFs. These findings demonstrate that TG2 activation is a Ca^2+^‐dependent event in nsPEF‐exposed cells and exerts negative effects on cell physiology.

**Abbreviations**
nsPEF: nanosecond pulsed electric field
PEF: pulsed electric field
TG: transglutaminase

Pulsed electric fields (PEFs) have unique actions on living organisms depending on their pulse duration. PEFs in the duration of milli‐ to microseconds generate membrane pores suited for macromolecule transfer 1, 2. Exposure of living cells to these PEFs is called electroporation and commonly used for DNA transfection and cancer chemotherapy 3, 4, 5. Nanosecond pulsed electric field (nsPEFs) are increasingly recognized to have biological actions distinct from electroporation. The pulse duration of nsPEFs is too short for generating membrane pores sufficiently large for macromolecule transfer. Thus, nsPEFs are not suited for electroporation‐based applications, such as DNA transfection. Instead, nsPEFs generate small membrane pores that permeate ions and water 6, 7, 8. Accordingly, exposure of living cells to nsPEFs causes the influx of extracellular Ca^2+^ and blebbing of the cell membrane, presumably due to ion imbalance 9, 10, 11.

In addition to ion permeation, nsPEFs induce various cellular reactions, including signal transduction 12, 13, 14, stress response 15, and cell death 16, 17. Intense nsPEFs induce either apoptotic or necrotic cell death, and cell‐type dependency of the cell death modes has been reported. For example, nsPEFs induce apoptosis in HL‐60 and Jurkat cells 16, 18, 19, whereas necrotic cell death is elicited in several cell lines, such as HeLa S3 20, 21. Previous studies suggested a critical role of Ca^2+^ in the induction of necrotic cell death by nsPEFs 11, 21. Pretreatment with a Ca^2+^ ionophore augmented the cytotoxic effects of nsPEFs on HeLa S3 cells, and the absence of extracellular Ca^2+^ suppressed the induction of necrosis in HeLa S3 cells but not that of apoptosis in Jurkat cells 21. Although these observations strongly suggest the importance of Ca^2+^ for cellular responses to nsPEFs, limited information is currently available regarding specific intracellular reactions that are affected by nsPEF‐induced Ca^2+^ influx.

Ca^2+^ exerts profound effects on a wide range of intracellular reactions, one of which is a post‐translational protein modification by transglutaminases (TGs), particularly by TG2 22, 23. TG family members catalyze transamidation, which is the conjugation of a glutamine residue in a protein with an amine donor 22, 23. When a lysine residue of another protein is used as an amine donor, TG‐mediated transamidation results in the formation of protein‐protein crosslinking (Fig. 1A), which leads to alterations in protein properties such as decreased solubility, restricted conformational flexibility, and resistance to proteolytic degradation 24, 25.

**Figure 1:**
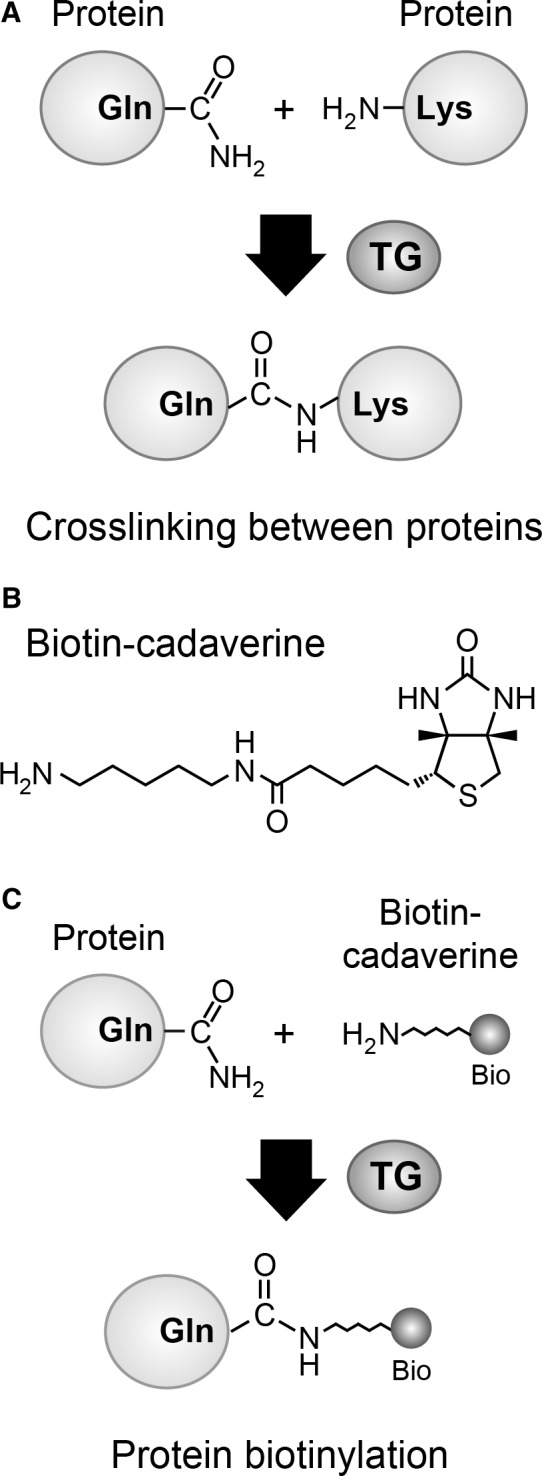
Protein modification by TG. (A) Protein‐protein crosslinking by TG. TG catalyzes the formation of a covalent bond between glutamine and lysine residues, resulting in crosslinking between two proteins. (B) Structure of biotin‐cadaverine, a cell‐permeating pseudosubstrate for TG. (C) TG‐mediated conjugation between a protein and biotin‐cadaverine. The biotin moiety of biotin‐cadaverine is shown as ‘Bio’. TG catalyzes the conjugation between a glutamine residue in a protein and the amine group of biotin‐cadaverine, yielding protein biotinylation.

The human TG family consists of nine members, and TG2 is a major member distributing almost all cell types in the human body 26. TG2 requires elevated intracellular Ca^2+^ levels for its enzymatic activity, and is therefore catalytically inert under normal physiological conditions owing to low intracellular Ca^2+^ 22, 23. Certain physiological processes increase intracellular Ca^2+^ to levels sufficient for the activation of TG2. In addition to a requirement of elevated intracellular Ca^2+^ levels, TG2 activity is affected by its subcellular localization, conformational states, and interacting proteins, all of which are determined in a cellular context‐dependent manner 26, 27, 28, 29. Under normal physiological conditions, TG2 is involved in various cellular processes, one of which is the control of cell death and survival. Depending on the cellular context, TG2 facilitates either cell death or survival 22, 28, 30.

In pathological situations, aberrant activation of TG2 has been frequently observed, particularly in neurodegenerative diseases, including Huntington disease and Parkinson disease 23, 24. Neurodegenerative diseases are characterized by aggregation of disease‐specific proteins, such as huntingtin in Huntington disease and α‐synuclein in Parkinson disease. Aggregate formation of these disease‐specific proteins is regarded as a primary cause of progressive loss of nerve cells, leading to pathogenesis. Under experimental conditions, TG2 has been demonstrated to promote aggregations of disease‐specific proteins by Ca^2+^‐dependent protein crosslinking 31, 32, 33. Although TG2 is not regarded as a primary cause of the onset of neurodegenerative diseases 24, protein crosslinking by dysregulated TG2 is known to promote neuropathology 23, 25. Collectively, TG2 plays critical roles in various cellular activities in both physiological and pathological situations 22, 23.

Considering the induction of Ca^2+^ influx by nsPEFs and the requirement of elevated Ca^2+^ for TG2 activity, we investigated the TG2 activation by nsPEFs. We utilized bitoin‐cadaverine, a cell‐permeable pseudosubstrate for TGs, and monitored the overall catalytic activity of TGs as conjugation of biotin‐cadaverine with cellular proteins. We observed that exposure of HeLa S3 cells to nsPEFs caused marked elevation of the TG activity in a Ca^2+^‐dependent manner. Depletion of the TG2 protein by RNA interference (RNAi) significantly suppressed the nsPEF‐induced TG activation and partially alleviated cytotoxic effects of nsPEFs. Collectively, these observations demonstrate that nsPEFs highly activate TG2 and suggest that massive protein‐protein crosslinking arises in nsPEF‐exposed cells and exerts negative effects on cell physiology.

## Materials and methods

### Cell culture and preparation of cell suspension

HeLa S3 cells were obtained from the American Type Culture Collection and were grown in α‐modified minimum essential medium (αMEM) supplemented with 10% fetal bovine serum (FBS, Corning Inc., Corning, NY, USA), 100 μg·mL^−1^ streptomycin, and 100 units·mL^−1^ penicillin. Cells were cultured under standard conditions at 37 °C in a humidified incubator containing 5% CO_2_.

For exposure to nsPEFs, cells were detached by treating with 1 mm EDTA dissolved in Dulbecco's phosphate‐buffered saline (D‐PBS) and suspended in αMEM containing heat‐inactivated FBS. Cells were collected by brief centrifugation and suspended in the same medium.

To analyze the effects of Ca^2+^, Ca^2+^‐free Dulbecco's modified Eagle's medium (Thermo Fisher Scientific, Waltham, MA, USA) and heat‐inactivated dialyzed FBS (Sigma‐Aldrich, St. Louis, MO, USA) were used in place of MEMα supplemented with regular FBS. From the dialyzed FBS, low molecular weight materials such as Ca^2+^ were removed by dialysis against 0.15 m NaCl with a cut‐off value of 10 000 Da. To prepare Ca^2+^‐containing medium, CaCl_2_ was added to Ca^2+^‐free medium at 1.8 mm.

### Treatment of cells with nsPEF exosure and UV irradiation

Exposure of cells to nsPEFs was performed as described previously 13, 15, 19. Briefly, cell suspension prepared as mentioned above (400 μL) was placed in an electroporation cuvette that had a pair of aluminum electrodes at a 4 mm‐distance (#5540, Thermo Fisher Scientific). Shots of nsPEFs at 1 Hz were generated using a pulsed power modulator (MPC3000S, Suematsu Electronics, Kumamoto, Japan) and applied to cells in an electroporation cuvette. Voltage waveforms of nsPEFs were monitored using a high voltage probe (P6015A, Tektronix, Beaverton, OR, USA) and a digital phosphor oscilloscope (TDS2012C, Tektronix). The average pulse width at half maximum was estimated to be ~ 80 ns 13, 15, 19.

For UV irradiation, cells were rinsed with D‐PBS and subsequently irradiated with 312 nm UV light using a BLX‐312 UV irradiator (Vilber Lourmat, Marne‐la‐Vallee, France). After the addition of prewarmed medium, cell culture was continued under the standard conditions and used for further experiments.

### Western blot analysis of cellular proteins conjugated to biotin‐cadaverine

Biotin‐cadaverine (also referred to as 5‐(biotinamido)pentylamine) was purchased from AAT Bioquest. Cell suspension was preincubated in medium containing 0.2 mm biotin‐cadaverine for 20 min at 37 °C to allow loading of biotin‐cadaverine. The cell suspension was exposed to nsPEFs and in turn diluted five‐fold in prewarmed medium containing the same concentration of biotin‐cadaverine. Whole cell lysates were prepared in SDS/PAGE sample buffer as described previously 21 and subjected to western blot analysis. Biotinylated proteins were detected using streptavidin conjugated with horseradish peroxidase (HRP; Wako Pure Chemical Industries, Osaka, Japan). Antibodies against Ku70 (loading control, Cell Signaling Technology, Danvers, MA, USA) and TG2 (GTX111702, GeneTex, Irvine, CA, USA) were used to detect respective proteins. Chemiluminescence was generated using a Super Signal West Pico reagent (Thermo Scientific) and detected using a ChemiDoc XRS Plus imaging system (Bio‐Rad, Hercules, CA, USA). Western blot images were quantified using imagelab software (Bio‐Rad). For the analysis of poly(ADP‐ribose) formation and caspase 3 cleavage, HeLa S3 cells were treated with nsPEF exposure or UV irradiation without biotin‐cadaverine preloading and then subjected to western blot analysis using antibodies against poly(ADP‐ribose; Clone 10H, Tulip Biolabs, West Point, PA, USA) and caspase 3 (Cell Signaling Technology).

### Immunofluorescence microscopy

Cells were fixed with 4% paraformaldehyde dissolved in D‐PBS for 30 min at 4 °C. After washing with D‐PBS, cells were treated with 0.1% Triton X‐100 in D‐PBS for 3 min and in turn incubated with 1% bovine serum albumin in D‐PBS for 15 min. Cells were reacted with avidin conjugated with fluorescein isothiocyanate (avidin‐FITC, Sigma‐Aldrich) and anti‐α‐tubulin rabbit polyclonal antibody (Abcam, Cambridge, UK) overnight at 4 °C. After washing with D‐PBS, cells were incubated with an anti‐rabbit antibody conjugated with Alexa 594 and mounted in Vectashield mounting medium (Vecror Laboratories, Burlingame, CA, USA) containing 4′,6‐diamidino‐2‐phenylindole (DAPI). Fluorescence was observed using an FV1200 laser scanning confocal microscope (Olympus, Tokyo, Japan) and analyzed with fluoview software (Olympus).

### RNA interference

Double‐stranded small interfering RNA (siRNA) corresponding to human TG2 mRNA with dTdT overhangs was synthesized by Nippon Gene (Toyama, Japan). The sequence of TG2 siRNA (sense strand) was as follows: 5′‐GAGCGAGAUGAUCUGGAAC‐3′. Double‐stranded negative control siRNA that does not exhibit significant sequence similarity to any human mRNA species was purchased from Nippon Gene (Toyama, Japan). Transfection of siRNA was conducted using 15 nm siRNA in media with a Lipofectamine RNAiMax transfection reagent (Thermo Fisher Scientific) according to the manufacturer's instruction. Cells were subjected to exposure to nsPEFs at 48 h after transfection.

### Measurement of cell viability

Cell viability was evaluated by the MTT method. An MTT reagent (3‐(4,5‐dimethylthiazol‐2‐yl)‐2,5‐diphenyltetrazolium bromide) was purchased from Dojindo Laboratories (Kumamoto, Japan) and was dissolved at 5 mg·mL^−1^ in D‐PBS. Cells (15 000) in a volume of 100 μL were incubated at 37 °C for 24 h after nsPEF exposure or UV irradiation. Thereafter, 10 μL of MTT solution was added to each well, and incubation was continued for 3 h. To dissolve formazan products, 100 μL solubilizing solution (10% SDS, 0.1 N HCl) was added, and incubation was continued overnight. Absorbance was measured at 570 nm using a microplate reader (iMark microplate reader, BioRad). Medium without cells was included in assays, and the measured value without cells was subtracted. Cell viability was expressed as the percentage of MTT values obtained with the untreated cells. Statistical analysis was performed using a Student's *t*‐test, and significance levels (*P* values) are indicated in the figures.

## Results

### Induction of covalent modifications of cellular proteins with biotin‐cadaverine by nsPEFs

Transglutaminases catalyzes a covalent modification of a glutamine residue of a protein with a variety of amine‐donor substrates 22. When a lysine residue of another protein is used as an amine donor, a TG‐catalyzed reaction yields a protein‐protein crosslinking between glutamine and lysine residues (Fig. 1A). The overall activity of cellular TGs can be measured using biotin‐cadaverine, which is a cell‐permeating pseudosubstrate for TG (Fig. 1B) 34. Catalytically active TG conjugates biotin‐cadaverine to glutamine residues of various cellular proteins, resulting in protein biotinylation (Fig. 1C) 34, which can be detected by avidin‐linked probes, such as streptavidin‐HRP for western blotting and avidin‐FITC for fluorescence microscopy.

**Figure 2:**
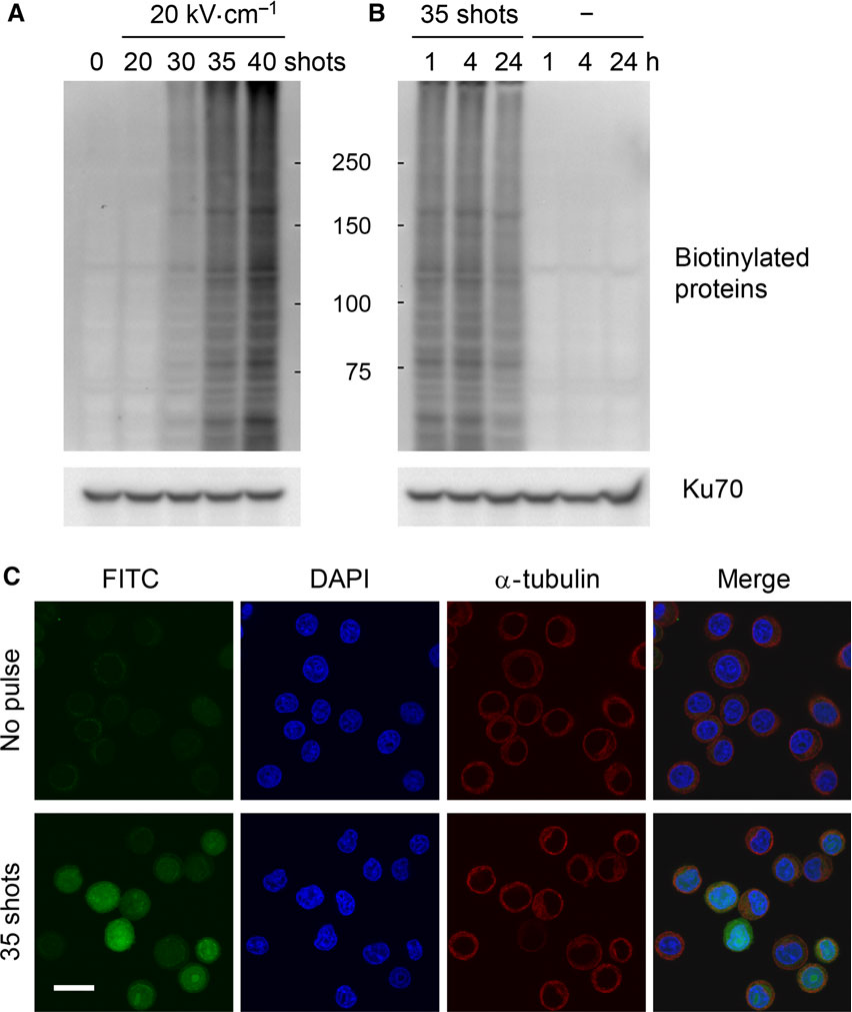
Induction of protein modifications with biotin‐cadaverine by nsPEFs. HeLa S3 cells were preincubated with 0.2 mm biotin‐cadaverine for 20 min and subsequently subjected to exposure to nsPEFs. (A) Indicated numbers of shots of nsPEFs at 20 kV·cm^−1^ were applied to HeLa S3 cells in the presence of biotin‐cadaverine. After 1 h incubation at 37 °C, the cells were subjected to western blot analysis for biotinylated proteins. Ku70 protein is shown as a loading control. Three independent experiments were performed to confirm the reproducibility, and a representative result is shown. (B) HeLa S3 cells were exposed to 35 shots of 20 kV·cm^−1^ nsPEFs and incubated for the indicated periods. Untreated cells (indicated as –) were included in the analysis. Biotinylated proteins and Ku70 were analyzed by western blotting as described in (A). Three independent experiments were performed to confirm the reproducibility, and a representative result is shown. (C) HeLa S3 cells exposed to nsPEFs (35 shots of 20 kV·cm^−1^) and untreated control cells were incubated at 37 °C for 1 h and subsequently costained with FITC‐streptavidin, anti‐α‐tubulin antibody, and DAPI. Three independent experiments were performed to confirm the reproducibility, and a representative result is shown. Bar, 20 μm.

To investigate the effects of nsPEFs on TG activity, HeLa S3 cells were loaded with biotin‐cadaverine, exposed to nsPEFs, and subjected to analyses of biotinylated proteins by western blotting and fluorescence microscopy (Fig. 2). Western blot analysis using streptavidin‐HRP demonstrated that nsPEFs induce massive protein biotinylation (Fig. 2A and 2B). Biotinylated proteins were detected as smears in the western blot, presumably because biotin‐cadaverine was conjugated to various cellular proteins of a broad range of molecular weights. Although 20 shots of 20 kV·cm^−1^ nsPEFs were insufficient for the induction of protein biotinylation, 30 and more shots of nsPEFs yielded massive protein biotinylation (Fig. 2A). nsPEF‐induced protein biotinylation was relatively persistent and detectable at 24 h after nsPEF exposure (Fig. 2B). We next performed fluorescence microscopy using FITC‐avidin and observed broad distribution of biotinylated proteins in the cytoplasm and the nucleus of nsPEF‐exposed cells (Fig. 2C). Cells that were loaded with biotin‐cadaverine but not exposed to nsPEFs did not exhibit detectable increases in protein biotinylation by western blotting or fluorescence microscopy, indicating that biotin‐cadaverine did not react with cellular proteins under unstimulated conditions. Collectively, these results demonstrate that the overall cellular TG activity was markedly elevated after nsPEF exposure.

### Ca^2+^‐dependent induction of protein modifications with biotin‐cadaverine by nsPEFs

**Figure 3:**
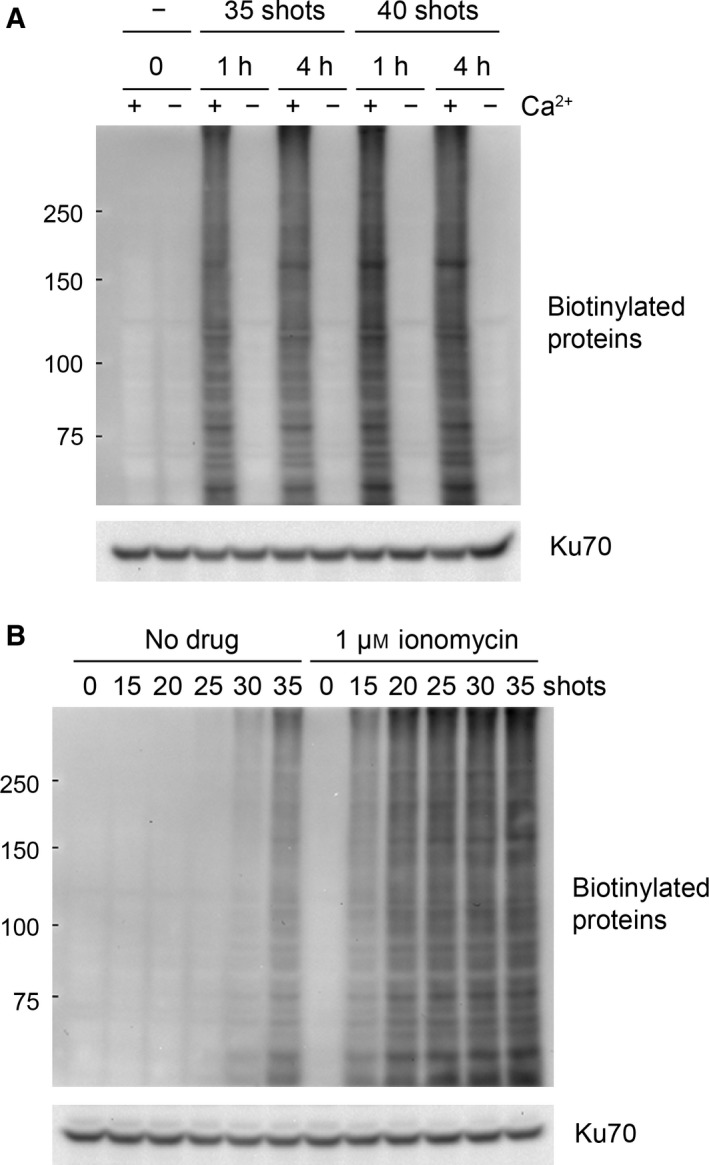
Ca^2+^‐dependent protein modifications with biotin‐cadaverine by nsPEFs. (A) HeLa S3 cells were loaded with biotin‐cadaverine in either Ca^2+^‐free (−) or 1.8 mm Ca^2+^‐containing (+) medium. The cells were exposed to 35 or 40 shots of 20 kV·cm^−1^ nsPEFs and incubated at 37 °C for 1 or 4 h. Western blot analysis of biotinylated proteins was performed as described in Fig. 2A. (B) HeLa S3 cells were loaded with biotin‐cadaverine in Ca^2+^‐containing medium The cells were exposed to the indicated shot numbers of 20 kV·cm^−1^ nsPEFs in the presence or absence of 1 μm ionomycin and subsequently incubated at 37 °C for 1 h. Western blot analysis of biotinylated proteins was performed as described in Fig. 2A.

Human cells have multiple TG family members that are differentially expressed depending on cell types 26. TG2 is a major member of the human TG family and ubiquitously distributes in almost all cell types in the human body 22, 26. TG2 is catalytically inert under normal physiological conditions, and its enzymatic activity requires elevated intracellular Ca^2+^ 22, 23. Because nsPEFs are well known to induce influx of extracellular Ca^2+^ 9, 10, 11, we next examined the importance of extracellular Ca^2+^ in the nsPEF‐induced conjugation of biotin‐cadaverine with cellular proteins. HeLa S3 cells loaded with biotin‐cadaverine were exposed to nsPEFs in the presence or absence of Ca^2+^ at a physiological concentration (1.8 mm), and protein biotinylation was analyzed by western blotting. We observed that the absence of extracellular Ca^2+^ totally abolished nsPEF‐induced protein biotinylation (Fig. 3A), suggesting that the influx of extracellular Ca^2+^ is critical for the elevation of TG activities by nsPEFs.

To further investigate the importance of Ca^2+^ in TG activation by nsPEFs, we repeated the experiments using the Ca^2+^ ionophore ionomycin and found that ionomycin augments protein biotinylation by nsPEFs (Fig. 3B). We observed that, in the presence of ionomycin, fewer shot numbers were sufficient for the induction of intense protein biotinylation. Ionomycin treatment without nsPEF exposure did not induce protein biotinylation, suggesting that both Ca^2+^ and nsPEF action are required for the elevation of the TG activity.

### Suppression of nsPEF‐induced biotin‐cadaverine conjugation by TG2 depletion

**Figure 4:**
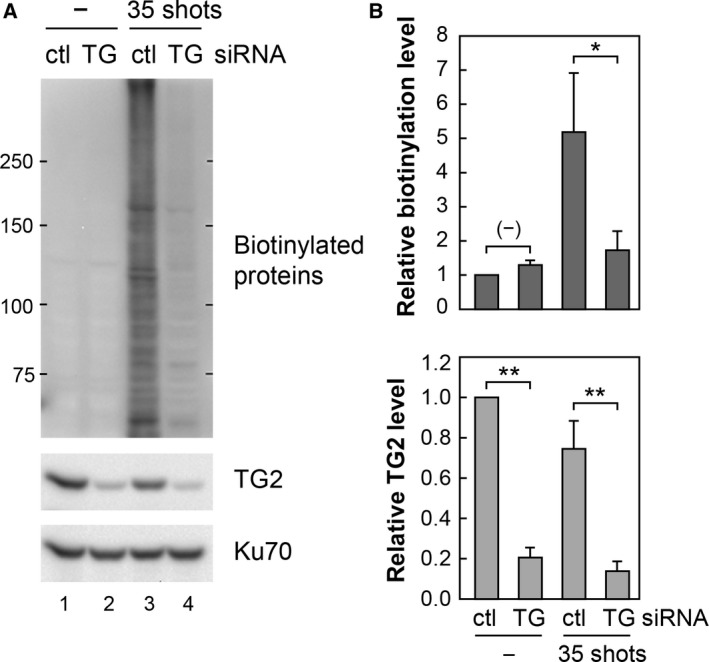
Suppression of nsPEF‐induced biotin‐cadaverine conjugation by TG2 RNAi. (A) HeLa S3 cells were transfected with either siRNA targeting TG2 (TG) or control siRNA (ctl). At 48 h after transfection, the cells were loaded with biotin‐cadaverine, exposed to 0 or 35 shots of 20 kV·cm^−1^ nsPEFs, and further incubated at 37 °C for 1 h. Western blotting was performed to analyze biotinylated proteins, TG2, and Ku70 (loading control). (B) Signal intensities of biotinylated proteins and TG2 in the western blot were quantified by densitometry and normalized relative to the signal of Ku70. The protein level in the control cells (transfected with control siRNA, no nsPEF exposure) was arbitrarily set to 1. Data represent the means ± standard deviations) from three independent experiments. Double asterisk (**) indicates *P* < 0.01, and a single asterisk (*) refers to *P* < 0.05.

Transglutaminases 2 is a predominant member of the human TG family, and its catalytic activity requires elevated Ca^2+^ levels 22. Given the dependence on Ca^2+^, we speculated that the nsPEF‐induced protein modifications with biotin‐cadaverine are mediated by TG2. To test this idea, TG2 siRNA was transfected into HeLa S3 cells to deplete the TG2 protein by RNAi. The siRNA‐transfected cells were loaded with biotin‐cadaverine, exposed to nsPEFs, and in turn subjected to western blot analysis for protein biotinylation and endogenous TG2 protein (Fig. 4A). Western blot signals for protein biotinylation and endogenous TG2 were quantified from three independent experiments (Fig. 4B). We observed that transfection of TG2 siRNA significantly reduced endogenous TG2 levels and concomitantly suppressed biotin‐cadaverine conjugation, indicating that TG2 is activated by nsPEFs and catalyzes covalent modifications of various cellular proteins with biotin‐cadaverine.

### Effects of TG2 depletion on the viability of nsPEF‐exposed cells

**Figure 5:**
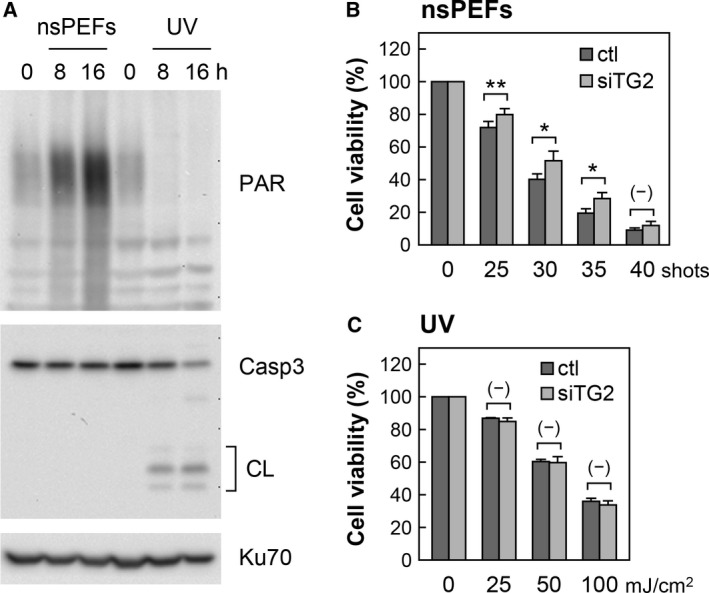
Effects of TG2 knockdown on cell viability after nsPEF exposure and UV irradiation. (A) Induction of necrosis by nsPEFs (30 shots at 20 kV·cm^−1^) and apoptosis by UV irradiation (100 mJ·cm^−2^) in HeLa S3 cells. Cells were treated with nsPEF exposure or UV irradiation and in turn subjected to western blot analysis of poly(ADP‐ribose) formation and caspase 3 cleavage. Cleaved species of caspase 3 are marked as CL. (B) Viability of TG2 depleted cells after nsPEF exposure. HeLa S3 cells were transfected with TG2 siRNA (siTG2) or control siRNA (ctl) and incubated for 48 h. The cells were exposed to the indicated shot numbers of 20 kV·cm^−1^ nsPEFs, and cell viability was measured at 24 h by the MTT method. Average values and standard deviations were calculated from eight independent experiments, and statistical differences were estimated. Double asterisk (**) indicates *P* < 0.01, and a single asterisk (*) refers to *P* < 0.05. No significant difference is shown by (–). (C) Viability of TG2 depleted cells after UV irradiation. TG2‐depleted and control cells were irradiated with 312 nm UV at the indicated doses. Cell viability at 24 h after UV irradiation was measured by the MTT method. Average values and standard deviations were calculated from three independent experiments, and there were no significant differences in cell viability between TG2 siRNA and control siRNA transfection.

Transglutaminases 2 plays complex roles in the control of cell death and survival, depending on cellular contexts and cell death‐inducing stimuli 22, 27, 30. To understand the biological significance of nsPEF‐induced TG2 activation, we examined the effect of TG2 activation on cell viability. First, we confirmed that nsPEFs and UV irradiation induce different modes of cell death, namely necrosis and apoptosis, respectively, in HeLa S3 cells (Fig. 5A). As reported in the previous studies 19, 21, we observed poly(ADP‐ribose) formation but not caspase 3 cleavage in nsPEF‐induced necrotic cells, whereas UV‐induced apoptotic cells exhibited caspase 3 cleavage but not poly(ADP‐ribose) formation (Fig. 5A). Next, we analyzed the viability of TG2‐depleted and control cells after nsPEF exposure or UV irradiation. MTT assays demonstrated that TG2 depletion by RNAi partially alleviated the cytotoxicity of nsPEFs (Fig. 5B), whereas that of UV irradiation was not affected by TG2 RNAi (Fig. 5C). These observations suggest that TG2 activation exerts a facilitative effect on nsPEF‐induced necrotic cell death in HeLa S3 cells.

## Discussion

Although nsPEFs have attracted much interest as a novel physical means for biological and medical applications 35, 36, their molecular mechanisms of action remain to be fully elucidated. nsPEFs are known to generate small pores on the cell membrane, which permeate small molecules, such as ions and water 6, 7, 8. Accordingly, nsPEFs have been shown to provoke the influx of extracellular Ca^2+^ 9, 10. In this study, we demonstrated that nsPEFs activate TG2, which is a predominant member of the human TG family and requires elevated intracellular Ca^2+^ for its catalytic activity. TG family members catalyze conjugation between glutamine and lysine residues of proteins, resulting in protein‐protein crosslinking. Although the occurrence of protein‐protein crosslinking in a cell is difficult to analyze, cellular TG activity can be monitored as conjugation of biotin‐cadaverine to cellular proteins, yielding protein biotinylation. As shown in Fig. 2, we observed intense biotinylation of a broad range of cellular proteins after nsPEF exposure, suggesting that massive protein‐protein crosslinking arise in nsPEF‐exposed cells. We speculate that overactivation of TG2 and consequent excessive protein‐protein crosslinking may have detrimental effects on cell physiology.

Previous studies have demonstrated that intense nsPEFs induce necrotic as well as apoptotic cell death in a cell type‐dependent manner 16, 18, 19, 20, 21. Exposure of HeLa S3 cells to nsPEFs causes necrosis, whereas apoptosis is induced in Jurkat cells by nsPEFs 19. Our previous study has shown that efficient induction of necrosis by nsPEFs requires the presence of extracellular Ca^2+^ at physiological concentrations, and that the absence of extracellular Ca^2+^ preserves the high viability of nsPEF‐exposed HeLa S3 cells 21. The induction of apoptosis by nsPEFs in Jurkat cells was largely independent of Ca^2+^ 21. Although these observations strongly suggest the importance of Ca^2+^ influx in nsPEF‐induced necrosis, limited information is currently available regarding how elevated Ca^2+^ affects intracellular reactions and cell viability. It has been speculated that elevation of intracellular Ca^2+^ by nsPEFs may exert profound effects on various cellular processes. For example, Ca^2+^ overload in mitochondria generally impairs ATP production and consequently results in low cellular energy status, frequently leading to necrotic cell death 37. Elevated Ca^2+^ is also known to activate certain types of proteases, such as calpain and cathepsin, both of which promote necrotic cell death 38. Furthermore, elevated Ca^2+^ causes production of reactive oxygen species (ROS), and in fact, a previous study demonstrated that nsPEFs generate ROS in a Ca^2+^‐dependent manner 39. The present study shows that TG2 depletion by RNAi partly relieves the cytotoxic effect of nsPEFs, indicating that TG2 activation is one of the Ca^2+^‐dependent deleterious reactions induced by nsPEFs. We speculate that the overall detrimental effects of elevated Ca^2+^ perturb cellular homeostasis and eventually lead to necrosis in nsPEF‐exposed cells.

Dysregulation of TG2 and its contribution to pathogenesis have been well documented in neurodegenerative diseases, such as Huntington disease 23, 24. Neurodegenerative diseases are characterized by the progressive loss of nerve cells accompanied by aggregate formation of disease‐specific proteins. In Huntington disease, the huntingtin protein with an extended polyglutamine tract forms insoluble aggregates that are toxic to nerve cells, thereby causing the pathogenesis. TG2 has been known to play a facilitative role in the aggregate formation and exacerbation of neurodegenerative diseases 24, 25. Perturbations in Ca^2+^ homeostasis frequently occur in various pathological situations 40 and cause aberrant elevation in TG2 catalytic activity 24. Protein‐protein crosslinking by TG2 promotes the formation of protein aggregates and renders these aggregates resistant to proteolytic degradation, thereby aggravating neurodegenerative diseases 23, 24, 25. In the case of nsPEF‐exposed cells, TG2 appears to catalyze crosslinking of various cellular proteins, as indicated by the smearing patterns of the western blot signals for biotinylated proteins (Figs 2 and 3). Protein‐protein crosslinking generally decreases solubility and intracellular mobility of proteins. Furthermore, protein‐protein crosslinking restricts conformational plasticity, thereby impeding protein functions, such as a protein‐protein interaction, substrate recognition, and catalysis. Massive protein‐protein crosslinking caused by nsPEFs seems to bring about deleterious effects on biological functions of various proteins and presumably contributes to the cytotoxic actions of nsPEFs.

In conclusion, we demonstrated that nsPEFs activate TG2 in a Ca^2+^‐dependent manner. TG2‐mediated massive protein modifications in nsPEF‐exposed cells appear to exert negative effects on protein functions and cell physiology. Because Ca^2+^ is involved in the control of diverse cellular processes, nsPEF‐induced Ca^2+^ influx should affect many cellular reactions, in addition to TG2 activation. Further investigation on Ca^2+^‐dependency of nsPEF‐induced cellular reactions would provide a better understanding of nsPEF actions and more effective biomedical applications of nsPEFs.

## Author contributions

KMY and KY conceived the project, performed the experiments, and wrote the manuscript.
